# Supplementary material for: First-in-human robotic supermicrosurgery using a dedicated microsurgical robot for treating breast cancer-related lymphedema: a randomized pilot trial
Source: Nat Commun. 2020 Feb 11;11:757. doi: 10.1038/s41467-019-14188-w (PMC7012819; doi:10.1038/s41467-019-14188-w)
Supplement: Supplementary file 1 — Supplementary Information [file 41467_2019_14188_MOESM1_ESM.pdf]

Supplementary Information

First-in-human robotic supermicrosurgery using a dedicated microsurgical robot for treating breast cancer-related lymphedema: a randomized pilot trial

van Mulken TJM, Schols RM, et al.

Supplementary Figure 1. Duration of the anastomoses in minutes

Supplementary Figure 2. Lymph-ICF scores

Supplementary Table 1. SAMS and UWOMSA scores of anastomoses performed

Supplementary Table 2. Patient comfort and surgeon performance during LVA procedure

Supplementary Table 3. Patient comfort and surgeon performance during LVA procedure

Supplementary Table 4. Patient comfort and surgeon performance during LVA procedure

Supplementary Figure 1. Duration of the anastomoses in minutes

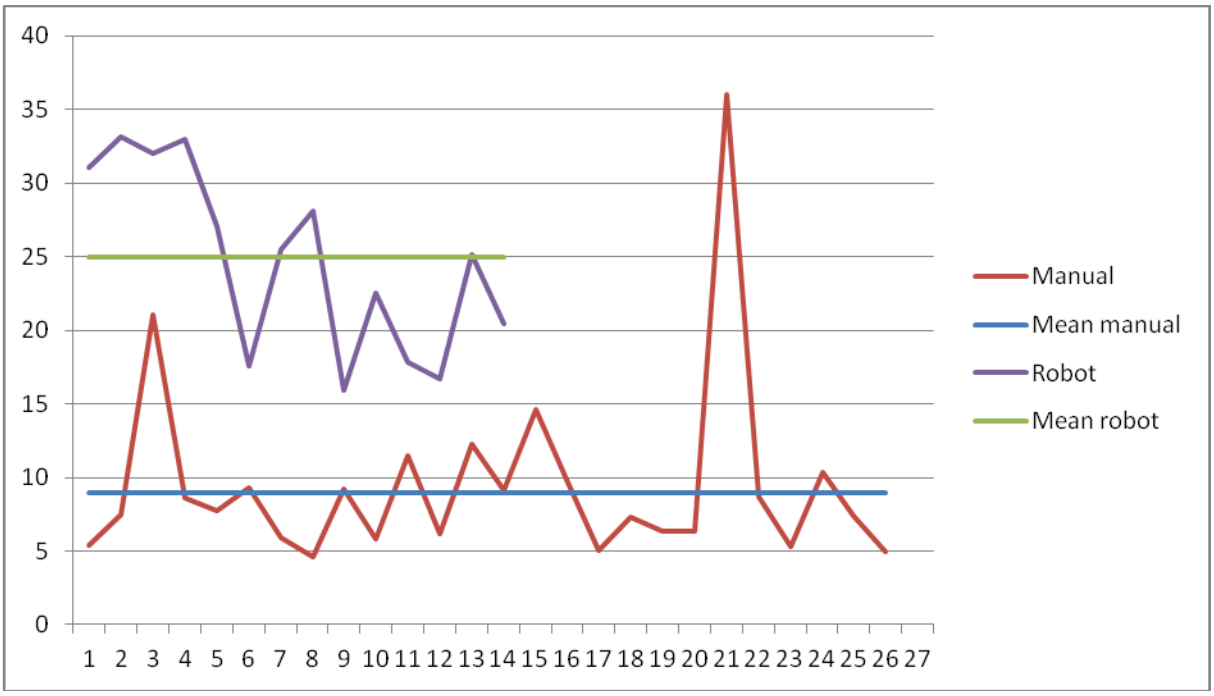

Duration of the anastomoses in minutes completed by robot-assistance versus the manual procedure, in consecutive order. Mean duration of respectively manual (n=14) and robotic (n=26) anastomoses are provided in the figure. Source data are provided as a Source Data file.

Supplementary Figure 2. Lymph-ICF scores

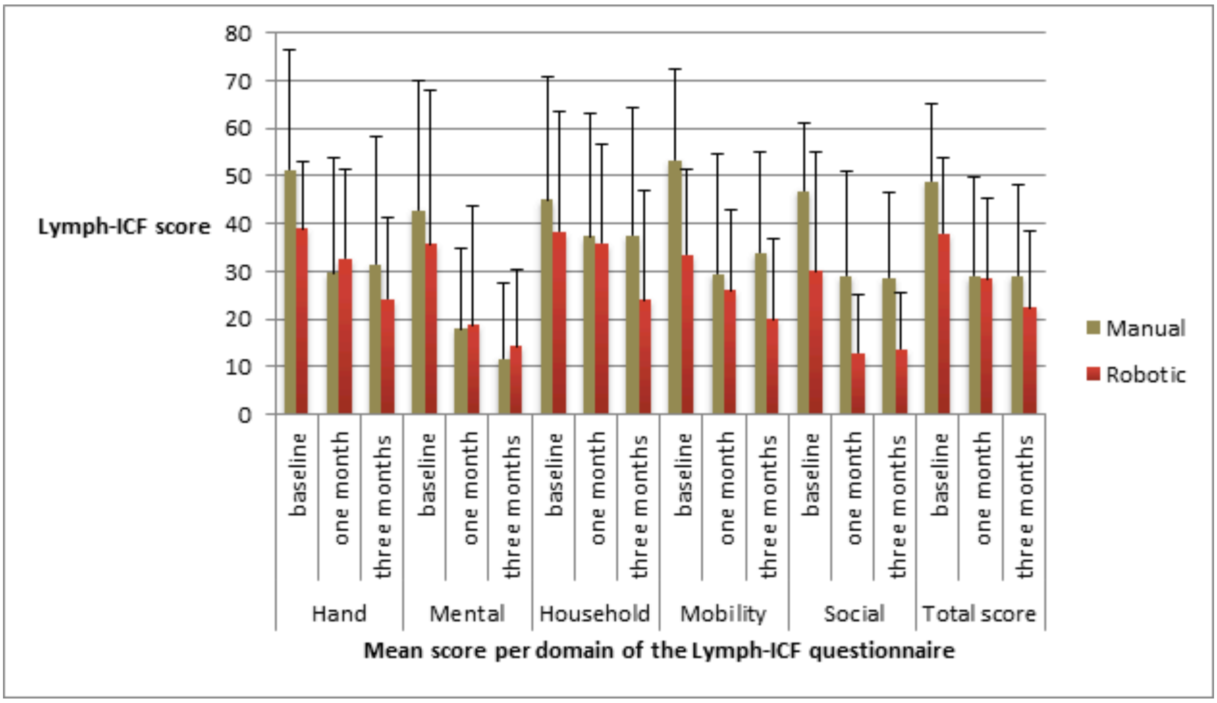

Mean lymph-ICF scores per domain and total score for the robotic and manual LVA group at baseline, one month and three months follow-up. Error bars of SD are included. Source data are provided as a Source Data file.

Supplementary Table 1. SAMS and UWOMSA scores of anastomoses performed

SAMS				UWOMSA		
		robotic (n=14)	manual (n=26)		robotic (n=14)	manual (n=26)
Dexterity	Steadiness	3.2±0.5	3.4±0.5	Preparation	3.5±.0.3	3.9±0.5
	Instrument handling	3.0±0.5	3.8±0.4	Suturing	3.2±0.4	3.9±0.4
	Tissue handling	3.2±0.5	3.8±0.4	Final product	3.4±0.2	4.0±0.5
Visuo-spatial ability	Dissection	3.4±0.6	4.0±0.4			
	Suture placement	3.5±0.5	4.1±0.3			
	Knot technique	3.8±0.5	4.3±0.3			
Operative flow	Steps	3.2±0.5	4.0±0.5			
	Motion	2.9±0.5	3.8±0.5			
	Speed	3.0±0.7	4.0±0.6			
Judgement	Irrigation	4.2±0.6	4.6±0.6			
	Patency test	2.4±1.6	3.8±0.6			
	Bleeding control	2.8±1.1	3.9±0.8			
Overall performance		3.0±0.7	4.0±0.5			
Indicative skill		3.0±0.6	4.0±0.5			

Data shown as average score of two readers

Mean ± SD of SAMS and UWOMSA scores. Source data are provided as a Source Data file.

Supplementary Table 2. Patient comfort and surgeon performance during LVA procedure

Group Statistics

	Surgery: hand or robot	N	Mean	Std. Deviation	Std. Error Mean
Comfort during LVA	hand	12	8,5833	1,10440	0,31881
	robot	8	8,0000	2,00000	0,70711
Performance surgeon during LVA	hand	12	3,75	0,754	0,218
	robot	8	3,13	0,641	0,227

Mean ± SD of patient comfort and surgeon performance during LVA respectively. Source data are provided as a Source Data file.

Supplementary Table 3. Patient comfort and surgeon performance during LVA procedure

Independent Samples Test

				t-test for Equality of Means						
				t	df	Sig. (2-tailed)	Mean Difference	Std. Error Difference	95% CI of the Difference	
									Lower	Upper
Comfort during LVA procedure	Equal variances assumed	2,205	0,155	0,843	18	0,411	0,58333	0,69236	-0,87126	2,03792
	Equal variances not assumed			0,752	9,876	0,470	0,58333	0,77566	-1,14789	2,31456
Performance surgeon during LVA procedure	Equal variances assumed	0,230	0,637	1,923	18	0,070	0,625	0,325	-0,058	1,308
	Equal variances not assumed			1,990	16,782	0,063	0,625	0,314	-0,038	1,288

Independent samples test of patient comfort and surgeon performance during LVA respectively. Source data are provided as a Source Data file.

Supplementary Table 4. Patient comfort and surgeon performance during LVA procedure

Statistics

		Comfort during LVA procedure	Performance surgeon during LVA procedure
N	Valid	20	20
	Missing	0	0
Std. Deviation		1,50525	0,761
Range		6,00	3
Minimum		4,00	2
Maximum		10,00	5

Statistics (SD, Range) of patient comfort and surgeon performance during LVA respectively. Source data are provided as a Source Data file.
