# Supplementary material for: First-in-human robotic supermicrosurgery using a dedicated microsurgical robot for treating breast cancer-related lymphedema: a randomized pilot trial
Source: Nat Commun. 2020 Feb 11;11:757. doi: 10.1038/s41467-019-14188-w (PMC7012819; doi:10.1038/s41467-019-14188-w)
Supplement: Supplementary file 3 — Reporting Summary [file 41467_2019_14188_MOESM3_ESM.pdf]

nature research

Corresponding author(s): Rutger M. Schols
Last updated by author(s): Oct 7, 2019

Reporting Summary

Nature Research wishes to improve the reproducibility of the work that we publish. This form provides structure for consistency and transparency in reporting. For further information on Nature Research policies, see Authors & Referees and the Editorial Policy Checklist.

Statistics

For all statistical analyses, confirm that the following items are present in the figure legend, table legend, main text, or Methods section.

n/a	Confirmed	
☐	☒	The exact sample size (*n*) for each experimental group/condition, given as a discrete number and unit of measurement
☐	☒	A statement on whether measurements were taken from distinct samples or whether the same sample was measured repeatedly
☐	☒	The statistical test(s) used AND whether they are one- or two-sided<br>*Only common tests should be described solely by name; describe more complex techniques in the Methods section.*
☐	☒	A description of all covariates tested
☒	☐	A description of any assumptions or corrections, such as tests of normality and adjustment for multiple comparisons
☐	☒	A full description of the statistical parameters including central tendency (e.g. means) or other basic estimates (e.g. regression coefficient) AND variation (e.g. standard deviation) or associated estimates of uncertainty (e.g. confidence intervals)
☐	☒	For null hypothesis testing, the test statistic (e.g. *F*, *t*, *r*) with confidence intervals, effect sizes, degrees of freedom and *P* value noted<br>*Give P values as exact values whenever suitable.*
☒	☐	For Bayesian analysis, information on the choice of priors and Markov chain Monte Carlo settings
☒	☐	For hierarchical and complex designs, identification of the appropriate level for tests and full reporting of outcomes
☒	☐	Estimates of effect sizes (e.g. Cohen's *d*, Pearson's *r*), indicating how they were calculated

Our web collection on statistics for biologists contains articles on many of the points above.

Software and code

Policy information about availability of computer code

Data collection	Data was collected in SPSS Statistics for Windows version 23.0 (IBM Corp., Armonk, NY, USA)
Data analysis	Statistical analyses were performed using SPSS Statistics for Windows version 23.0 (IBM Corp., Armonk, NY, USA)

For manuscripts utilizing custom algorithms or software that are central to the research but not yet described in published literature, software must be made available to editors/reviewers. We strongly encourage code deposition in a community repository (e.g. GitHub). See the Nature Research guidelines for submitting code & software for further information.

Data

Policy information about availability of data

All manuscripts must include a data availability statement. This statement should provide the following information, where applicable:

- Accession codes, unique identifiers, or web links for publicly available datasets
- A list of figures that have associated raw data
- A description of any restrictions on data availability

The authors declare that all data supporting the findings of this study are available within the paper and its supplementary in formation. Source Data Files are provided.

Field-specific reporting

Please select the one below that is the best fit for your research. If you are not sure, read the appropriate sections before making your selection.

☒ Life sciences ☐ Behavioural & social sciences ☐ Ecological, evolutionary & environmental sciences

Life sciences study design

All studies must disclose on these points even when the disclosure is negative.

Sample size	This was a pilot study, sample size calculations are not performed in pilot studies. The data obtained in this study will provide information for future sample size calculations in large studies.
Data exclusions	No data were excluded.<br>Two subjects from the robot-assisted group received manual LVA due to perioperative technical set-up error of the MSR. Analyses were based on the final type of surgery conducted.
Replication	No attempts were made to replicate this study. This study was a pilot study aimed to study the feasibility of robot-assistance in completing supermicrosurgical anastomosis. This concerns a first-in-human study using a newly developed microsurgical robot.
Randomization	Block randomization (block size 4) by a computer generated list was used to allocate the subjects.
Blinding	Data collection: the use of the microsurgical robot made double blinding not possible, neither for the surgeon, nor for the patient as the procedures were wide-awake under local anesthesia.<br>Data analysis: two microsurgeons, blinded for type of surgery, independently scored the quality of each anastomosis (n=40), using the Structured Assessment of Microsurgery Skills (SAMS) and University of Western Ontario Microsurgical Skills Acquisition Instrument (UWOMSA) scoring methods.

Reporting for specific materials, systems and methods

We require information from authors about some types of materials, experimental systems and methods used in many studies. Here, indicate whether each material, system or method listed is relevant to your study. If you are not sure if a list item applies to your research, read the appropriate section before selecting a response.

Materials & experimental systems

n/a	Involved in the study	
☒	☐	Antibodies
☒	☐	Eukaryotic cell lines
☒	☐	Palaeontology
☒	☐	Animals and other organisms
☐	☒	Human research participants
☐	☒	Clinical data

Methods

n/a	Involved in the study	
☒	☐	ChIP-seq
☒	☐	Flow cytometry
☒	☐	MRI-based neuroimaging

Human research participants

Policy information about studies involving human research participants

Population characteristics	Eligible patients were female adults ≥ 18 years old who suffered from unilateral early stage lymphedema of the arm (Stage 1 and 2 of the International Society of Lymphology (ISL) classification, mild, persistent or fibrotic lymphedema) after breast cancer treatment with axillary lymph node surgery and/or radiotherapy, and at least underwent three months of complex decongestive therapy without symptoms alleviation.
Recruitment	Females suffering from unilateral breast cancer-related lymphedema (BCRL) referred to the lymphedema outpatient clinic at Maastricht University Medical Center (MUMC) were invited to participate in this randomized pilot study.
Ethics oversight	Ethical approval was obtained from the institutional review board (IRB) of the academic hospital Maastricht/Maastricht University (Maastricht, the Netherlands).

Note that full information on the approval of the study protocol must also be provided in the manuscript.

Clinical data

Policy information about clinical studies

All manuscripts should comply with the ICMJE guidelines for publication of clinical research and a completed CONSORT checklist must be included with all submissions.

Clinical trial registration	The study was registered at the Netherlands Trial Register, with study number NTR6291.<br>https://www.trialregister.nl/trial/6291
Study protocol	The full study protocol is available at CCMO register.<br>https://www.toetsingonline.nl/to/ccmo_search.nsf/fABRpop?readform&unids=ED401FE8102C8EC3C125813300151DE1.

Data collection	The following patient characteristics were obtained:<br>- age, BMI, ISL stage, smoking status at baseline<br>- daily use of compression garment and manual lymph drainage at baseline, one month and three months post-surgery<br>- Lymph-ICF total score and UEL index of the affected arm at baseline, one month and three months post-surgery<br>Duration of the surgery was registered<br><br>At the end of each operation the patients´ overall convenience was assessed using a ten point VAS-score. The higher the score, the more convenience the patient had experienced during the surgery. Also the surgeons´ satisfaction with the procedure was assessed using a five point score. The higher the score, the higher the satisfaction of the surgeon.<br><br>Quality of the anastomosis was scored independently and blinded for type of surgery by two experienced microsurgeons using the validated SAMS method and UWOMSA score
Outcomes	- Primary outcome regarding the surgical procedure was quality of the anastomosis.<br>- Secondary outcomes included duration of the surgery, problems or technical errors during the procedure, postoperative complications and adverse events, Lymph-ICF total score (i.e. quality of life), UEL index of the affected arm (i.e. arm volume), patients´ convenience during the surgery and surgeons´ satisfaction of the procedure.<br><br>Because of the small sample size in both groups, the independent student's t-test and the Mann-Whitney U test were both evaluated for differences between groups for the variables age, BMI, UEL index of the affected arm and lymph-ICF total score. Differences per patient (percentage) were calculated for UEL index of the affected arm and Lymph-ICF total score between baseline and three months post-surgery.<br><br>In addition, linear mixed model analyses with an unstructured covariance structure for repeated measures were used to evaluate whether intervention (robot-assisted or manual LVA) influenced lymph-ICF total score or UEL index of the affected arm over time (one month or three months post-surgery, after correction for baseline differences).<br><br>For the quality of the anastomosis assessed using SAMS and UWOMSA, interreader reliability of two readers was analyzed for the mean SAMS score per reader for all items and UWOMSA score per domain using ICC with a two-way random effect model with absolute agreement, single measures. Reliability was rated according to Landis et al.; <0.00 poor, 0.00-0.20 slight, 0.21-0.40 fair, 0.41-0.60 moderate, 0.61-0.80 substantial, 0.81-1.00 almost perfect 30. SAMS and UWOMSA were further analyzed based on the average scores of both readers.<br><br>Differences in duration of anastomosis, and SAMS and UWOMSA score of the anastomoses between robot-assisted or manual LVA were analyzed using an independent-samples t-test. As sensitivity analyses, these differences were also analyzed using linear mixed models with surgery type as fixed factor and a random intercept on patient level to account for the clustering of anastomoses within patients.
