# Supplementary material for: First-in-human robotic supermicrosurgery using a dedicated microsurgical robot for treating breast cancer-related lymphedema: a randomized pilot trial
Source: Nat Commun. 2020 Feb 11;11:757. doi: 10.1038/s41467-019-14188-w (PMC7012819; doi:10.1038/s41467-019-14188-w)
Supplement: Supplementary file 4 — Source Data [file 41467_2019_14188_MOESM4_ESM.pdf]

LVA (0=hand/1=robot)	Patient comfort	Performance surgeon
1	7	3
0	7	4
1	7	4
0	9	3
0	10	4
1	4	3
0	9	4
1	9	3
1	9	3
0	8	4
0	7,5	4
1	8	2
0	7	3
1	10	4
1	10	3
0	10	5
0	8	4
0	9	2
0	10	4
0	8,5	4
